# LC–MS-based serum metabolomics reveals distinct metabolic signatures in patients with intracerebral Hemorrhage

**DOI:** 10.3389/fneur.2026.1795803

**Published:** 2026-07-02

**Authors:** Wei Zheng, Wei Wang, Xiaohui Lu

**Affiliations:** People’s Hospital of Bayingol Mongolian Autonomous Prefecture, Kuerle, China

**Keywords:** intracerebral hemorrhage, LC–MS, metabolites, metabolome, serum

## Abstract

**Introduction:**

Intracerebral hemorrhage (ICH) is a severe neurological disease with high mortality and disability, profoundly affecting patients’ neurological function, daily activities, and quality of life. This study aimed to characterize the serum metabolic profile of patients with ICH and identify potential metabolic biomarkers associated with disease pathogenesis.

**Methods:**

Liquid chromatography–mass spectrometry (LC–MS) was employed to systematically analyze serum metabolite profiles and class distributions in 20 patients with and without ICH. Data quality was evaluated using quality control samples, while orthogonal partial least squares-discriminant analysis (OPLS-DA), differential metabolite analysis, KEGG pathway enrichment, Human Metabolome Database (HMDB), Metabolite Set Enrichment Analysis (MSEA), and receiver operating characteristic (ROC) analyses were performed.

**Results:**

A total of 3,178 metabolites were identified. In patients with ICH, benzene and substituted derivatives were the most abundant metabolite class (15.63%), followed by organic acids (12.47%), amino acids and their metabolites (12.41%), and heterocyclic compounds (12.07%). Quality assessment demonstrated low variability in control samples (CV < 0.3), and OPLS-DA showed significant separation between the ICH and control groups (*p* < 0.01). Differential expression analysis revealed increased levels of benzene and substituted derivatives and organic acids, accompanied by decreased amino acids and lipid metabolites. KEGG pathway enrichment indicated significant involvement of linoleic acid, α-linolenic acid, arachidonic acid, retrograde endocannabinoid, choline metabolism in cancer, and glycerophospholipid metabolism. HMDB and MSEA analyses further demonstrated associations between differential metabolites and multiple metabolic diseases and physiological or pathological states. ROC analysis showed excellent diagnostic performance for several metabolites, with Dibutyl phthalate (AUC = 0.980), Octadecanamide (AUC = 0.960), Hypoxanthine (AUC = 0.840), and Lenticin (AUC = 0.810).

**Discussion:**

These findings demonstrate distinct alterations in the serum metabolomic profile of patients with ICH and provide new insights into the metabolic mechanisms underlying ICH. The identified differential metabolites may serve as promising biomarkers for the diagnosis and investigation of ICH.

## Introduction

Intracerebral hemorrhage (ICH) is an acute neurological disorder characterized by the rupture of a cerebral blood vessel which can lead to massive bleeding into the brain parenchyma and surrounding tissues that can result in neurological dysfunction (1, 2). Its main causes include arteriosclerosis, cerebral aneurysms, cerebral vascular malformations and brain trauma (3). As one of the most lethal types of stroke, ICH carries an extremely high mortality and disability rate with a mortality rate exceeding 50% (4). And it is the second leading cause of stroke-related death worldwide. Most patients die within the first month of onset, while survivors often suffer varying degrees of neurological impairment and multiple complications, such as recurrent cerebral hemorrhage, epilepsy, cognitive impairment, dementia, other neurological and non-neurological conditions (3).

Early detection and assessment of ICH are crucial for clinical intervention. Currently, conventional imaging modalities primarily include Computed Tomography (CT) and Magnetic Resonance Imaging (MRI) (5, 6). However, these methods may not be able to detect hematomas in patients with early ICH, especially those with small or unusually located hematomas (7). Moreover, imaging studies lack molecular insights into the pathological process and therefore cannot effectively predict the risk of hematoma progression or secondary brain injury. Therefore, researchers have attempted to use biomarkers in blood and cerebrospinal fluid to reflect neurological injury and inflammation, such as neurofilament (NF), S100 calcium binding protein B (S100B), creatine kinase isoenzyme (CK-BB), C-reactive protein (CRP) and interleukin-6 (IL-6) (8, 9, 10). But these biomarkers generally suffer from insufficient sensitivity, complex dynamic changes, and a lack of standardized testing protocols that can significantly limit their value in the early detection and monitoring of ICH.

In recent years, the development of metabolomics has provided new tools for a deeper understanding of the molecular mechanisms of disease (11, 12). Comprehensive analysis of metabolite profiles in blood or brain tissue can reveal abnormal metabolic pathways associated with disease and identify potential biomarkers which can provide a basis for personalized treatment (13). Disturbances in cholesterol and phospholipid metabolism are believed to contribute to the pathogenesis of Alzheimer’s disease (14). Serum levels of pyruvate, citric acid cycle-related metabolites and fatty acid metabolites in diabetic patients have been shown to be important indicators for predicting the onset of the disease and the risk of complications (15). Abnormalities in lipid metabolism, amino acid metabolism and organic acid metabolism have been shown to be closely associated with cerebrovascular disease and neurological damage (16). Furthermore, dynamic changes in metabolite levels can be used to assess disease progression or therapeutic efficacy, providing a quantitative basis for clinical diagnosis and treatment.

Currently, systematic studies of the serum metabolomics profiles of patients with ICH remain limited. This study compared the serum metabolite profiles and class distributions of 20 patients with and without ICH using liquid chromatography-mass spectrometry (LC–MS). Combined with multidimensional statistical analysis and pathway enrichment analysis, this study revealed metabolic abnormalities and potential biomarkers specific to ICH. This study not only contributes to a deeper understanding of the molecular mechanisms of ICH but also provides new insights and evidence for its early diagnosis, risk assessment, and targeted intervention.

## Materials and methods

### Participants

The subjects of this study were 10 patients with ICH (A001-A010) and 10 normal patients without ICH (B001-B010) from People’s Hospital of Bayingol Mongolian Autonomous Prefecture. The patients with ICH served as the experimental group and the patients without ICH served as the control group (Supplementary Table S1). Inclusion criteria of this study: (1) Age 18–65 years. (2) Patients with ICH needed to be confirmed by clinical characteristics and morphological examination. (3) Written informed consent was signed and blood samples were collected for metabolomics analysis. Exclusion criteria of this study: (1) History of acute or chronic infection within 2 weeks before sampling. (2) Pregnant or lactating women. (3) Use of drugs that affect metabolite profiles (such as glucocorticoids and chemotherapy drugs) within 1 month before sampling. (4) Patients with severe liver, kidney, or heart failure or malignant tumors. (5) Patients with mental disorders or other conditions that may affect compliance and cooperation. This study protocol has been approved by the Ethics Committee of Xinjiang Bayingol People’s Hospital (Approval Number: BZRMYY-LCYJ-2024-49). All patients signed informed consent forms.

### Detection of metabolites

All participants were required to fast for more than 8 h prior to blood collection. Approximately 5 mL of peripheral venous blood was collected using a disposable vacuum tube via venipuncture. Samples were allowed to clot at room temperature for 30 min, followed by centrifugation at 3000 × g for 10 min at 4 °C. The resulting serum was carefully transferred using RNase/DNase-free pipette tips into 1.5 mL enzyme-free cryogenic tubes and stored at −80 °C until analysis. This study used pooled quality control (QC) samples throughout the analysis to monitor instrument stability and data reproducibility, and did not add exogenous internal standards.

Frozen serum samples were thawed on ice until no visible ice remained, and vortex-mixed for 10 s. A 300 μL aliquot of extraction solvent (20% acetonitrile in methanol) was added to each 100 μL serum sample and vortexed for 3 min. The mixture was centrifuged at 12,000 × g for 10 min at 4 °C, and 200 μL of supernatant was transferred into a new tube. Samples were precipitated and purified at −20 °C for 30 min, followed by a second centrifugation at 12,000 × g for 3 min at 4 °C. Finally, 180 μL of supernatant was transferred into an autosampler vial with an insert for LC–MS/MS analysis.

Chromatographic separation (Japan, Shimadzu, LC-30A) was performed on a Waters ACQUITY Premier HSS T3 column (1.8 μm, 2.1 × 100 mm) installed on a Shimadzu LC-30A UPLC system. The mobile phase consisted of 0.1% formic acid in water (A) and 0.1% formic acid in acetonitrile (B). The column temperature was maintained at 40 °C, with a flow rate of 0.4 mL/min and an injection volume of 4 μL. The gradient program was as follows: 0.0 min 95% A/5% B, 2.0 min 80% A/20% B, 5.0 min 40% A/60% B, 6.0–7.5 min 1% A/99% B, 7.6–10.0 min re-equilibration to 95% A/5% B. Mass spectrometric detection was performed using an AB TripleTOF 6,600 system (Foster City, United States, SCIEX, TripleTOF) in both positive (ESI^+^) and negative (ESI^−^) ion modes, with a total run time of 10 min for MS1/MS2 acquisition. Ion spray voltage: ESI^+^ 5,000 V, ESI^−^ −4,000 V; ion source temperature: ESI^+^ 550 °C, ESI^−^ 450 °C; nebulizer/heater gas/curtain gas pressures: 50/60/35 psi. Declustering potential (DP): ESI^+^ 60 V, ESI^−^ −60 V. Collision energy (CE): MS1 ± 10 V, MS2 ± 30 V with a collision energy spread of 15 V. Mass scan range: MS1 m/z 50–1,000, MS2 m/z 25–1,000. Accumulation time: MS1 0.2 s and MS2 0.04 s, top 18 precursor ions were selected for fragmentation with dynamic exclusion after three occurrences for 3 s. The data of this study has been uploaded to the Metabolights database.^1^

### Metabolite annotation and data preprocessing

Raw LC–MS/MS data acquired from the AB TripleTOF 6,600 platform were converted into mzXML format using ProteoWizard MSConvert software (version 3.0) (17). The converted files were imported into R software (version 4.3.1) for peak detection, retention time correction, peak alignment and feature extraction using the XCMS package (version 3.18.0) (18). Peak picking was performed with the centWave algorithm using the following parameters: ppm = 15, peakwidth = c(5,30), snthresh = 10, mzdiff = 0.01, and prefilter = c(3,100). Retention time correction was conducted using the obiwarp method, peak grouping was performed with bw = 5 and mzwid = 0.015.

After peak alignment, metabolite features detected in less than 50% of QC samples or with coefficient of variation (CV) values >30% in QC samples were excluded to improve data reliability. Missing values were imputed using the half-minimum method. All 20 study samples were analyzed in a single analytical batch, with QC samples injected at the beginning of the run and subsequently every five samples. Signal drift correction and normalization were performed using QC-based robust LOESS signal correction (QC-RLSC) followed by Pareto scaling prior to multivariate statistical analysis. Metabolite annotation was performed based on accurate precursor mass, isotope distribution, retention time, and MS/MS fragmentation spectra by matching against with Human Metabolome Databas (HMDB) (19), METLIN (20), MassBank (21) and Kyoto Encyclopedia of Genes and Genomes (KEGG) (22). The mass error tolerance for precursor ion matching was set to <10 ppm, while MS/MS fragment ion matching tolerance was controlled within 0.02 Da. Metabolite identification confidence was assigned according to the Metabolomics Standards Initiative (MSI) reporting levels (23). Level 1a (*n* = 183) was verified using retention time, accurate mass, and MS/MS matching with authentic reference standards, Level 1b (*n* = 359) was confirmed via accurate mass and MS/MS spectral library matching without retention time alignment, Level 2 (*n* = 219) and Level 3 (*n* = 2,260) were putatively annotated/characterized based on database matching of MS/MS spectra and compound class spectral similarity, while Level 4 (*n* = 414) represented unknown features with no corresponding database matches (Supplementary Table S2).

After normalization, serum metabolomics data were subjected to unsupervised dimensionality reduction using principal component analysis (PCA) (24) in the R package to reveal underlying phenotypic characteristics and intergroup differences between patients with ICH (Group A) and those without ICH (Group B). This study also performed hierarchical clustering analysis (HCA) on the standardized metabolite abundances to identify overall differences in metabolite expression patterns between different groups.

### Orthogonal partial least squares discriminant analysis

To further compare the overall differences in serum metabolomics between patients with and without ICH, this study used orthogonal partial least squares discriminant analysis (OPLS-DA). Metabolite data were log-transformed and normalized using Pareto scaling before statistical analysis using R software. OPLS-DA models were constructed and visualized using the *ropls* package (25). Q^2^ was estimated by 7-fold cross-validation, in which the dataset was repeatedly divided into seven subsets. Each subset was held out in turn as a test set while the remaining data were used to fit the model, and the average prediction error across all held-out subsets was used to compute Q^2^. Model robustness was assessed using 200 permutation tests. In the permutation test, class labels were randomly permuted 200 times and the resulting R^2^Y and Q^2^ distributions were compared against the original model values, the model was considered valid when all permuted statistics fell below the original values (*p* < 0.005). The statistical significance of group separation in the OPLS-DA score plot was evaluated using cross-validated ANOVA (CV-ANOVA), with *p* < 0.01 considered statistically significant (26). Variable Importance in Projection (VIP) values were calculated from the OPLS-DA model, with a VIP > 1 serving as a screening threshold to identify key metabolites.

### Identification of differential metabolites

Serum metabolomics data were peak-extracted, aligned and normalized before statistical analysis using R software. Univariate analysis used the *limma* (27) package to calculate log₂(FC) and *p*-values, with the Benjamini–Hochberg method for FDR correction. For data not conforming to a normal distribution, the Wilcoxon rank sum test in the stats package was used. The screening criteria for differentially expressed metabolites were set as VIP > 1.0, adjusted *p*-value < 0.05 and |log₂FC| > 1.0. Volcano plots were created using the *ggplot2* (28) package for visualization, and metabolite classification statistics and subsequent functional annotation analysis were performed using the *MetaboAnalystR* (29) package.

### Receiver operating characteristic analysis of differential metabolites

To evaluate the diagnostic performance of the identified differential metabolites, receiver operating characteristic (ROC) curve analysis was performed using the pROC package (version 1.18.5) in R software (30). The area under the ROC curve (AUC) was calculated as a measure of diagnostic accuracy. Ninety-five percent confidence intervals (95% CIs) for AUC values were estimated using the nonparametric DeLong method. The optimal diagnostic cutoff value for each metabolite was determined using the Youden index (J = sensitivity + specificity − 1), and the corresponding sensitivity and specificity values were recorded. ROC curves were visualized using the ggplot2 package (28).

### Enrichment analysis

KEGG pathway enrichment analysis of differentially expressed metabolites was performed using the enricher() function in the R package *clusterProfiler* (v4.6.2) (31) with parameters: pvalueCutoff = 0.05, qvalueCutoff = 0.2, minGSSize = 10, maxGSSize = 500. Gene/metabolite IDs were then converted to a readable format using setReadable(org. Hs.eg.db, keyType = “KEGG”). Based on the KEGG analysis results, a metabolic pathway regulatory network was constructed and visualized using *igraph* (v1.5.1) (32) and MetaboAnalystR (v3.2.0) (29). *igraph* was used for network topology drawing, while MetaboAnalyst was used for node and edge attribute annotation and interactive visualization. Furthermore, differentially expressed metabolites were mapped to the HMDB (19), and enrichment analysis for metabolic disease associations was performed using the msetEnrichment() module in *MetaboAnalystR* (29). This module is based on the Metabolite Set Enrichment Analysis (MSEA) method. It explores the potential association between differential metabolites and human metabolic diseases by comparing them with known metabolite collection libraries (including disease-related metabolite sets) to evaluate their enrichment in specific physiological and pathological states (parameter settings: pvalueCutoff = 0.05, FDR = TRUE).

## Results

### Serum metabolite profile characteristics and category distribution in patients with and without cerebral hemorrhage

This study included 20 participants, divided into an ICH group (ICH group, *n* = 10) and a non-ICH control group (Control group, *n* = 10). In the ICH group, there were 5 males (50%) and 5 females (50%), with a mean age of 55.70 ± 10.58 years, a BMI of 26.15 ± 1.56 kg/m^2^, a systolic blood pressure (SBP) of 166.00 ± 13.43 mmHg, a diastolic blood pressure (DBP) of 99.90 ± 6.30 mmHg, a fasting blood glucose (FBG) of 6.36 ± 1.50 mmol/L, 8 patients (80%) had a history of hypertension and 6 patients (60%) had a history of diabetes. In the control group, there were 4 males (40%) and 6 females (60%), with a mean age of 51.60 ± 13.96 years, a BMI of 24.10 ± 2.30 kg/m^2^, a SBP of 126.30 ± 8.17 mmHg, a DBP of 78.50 ± 8.00 mmHg and a FBG of 5.22 ± 0.92 mmol/L. One patient (10%) had a history of hypertension, and two patients (20%) had a history of diabetes. Compared with the control group, the ICH group showed higher blood pressure, higher BMI and a higher prevalence of hypertension and diabetes, indicating a significant difference in metabolic status between the two groups.

**Figure 1:**
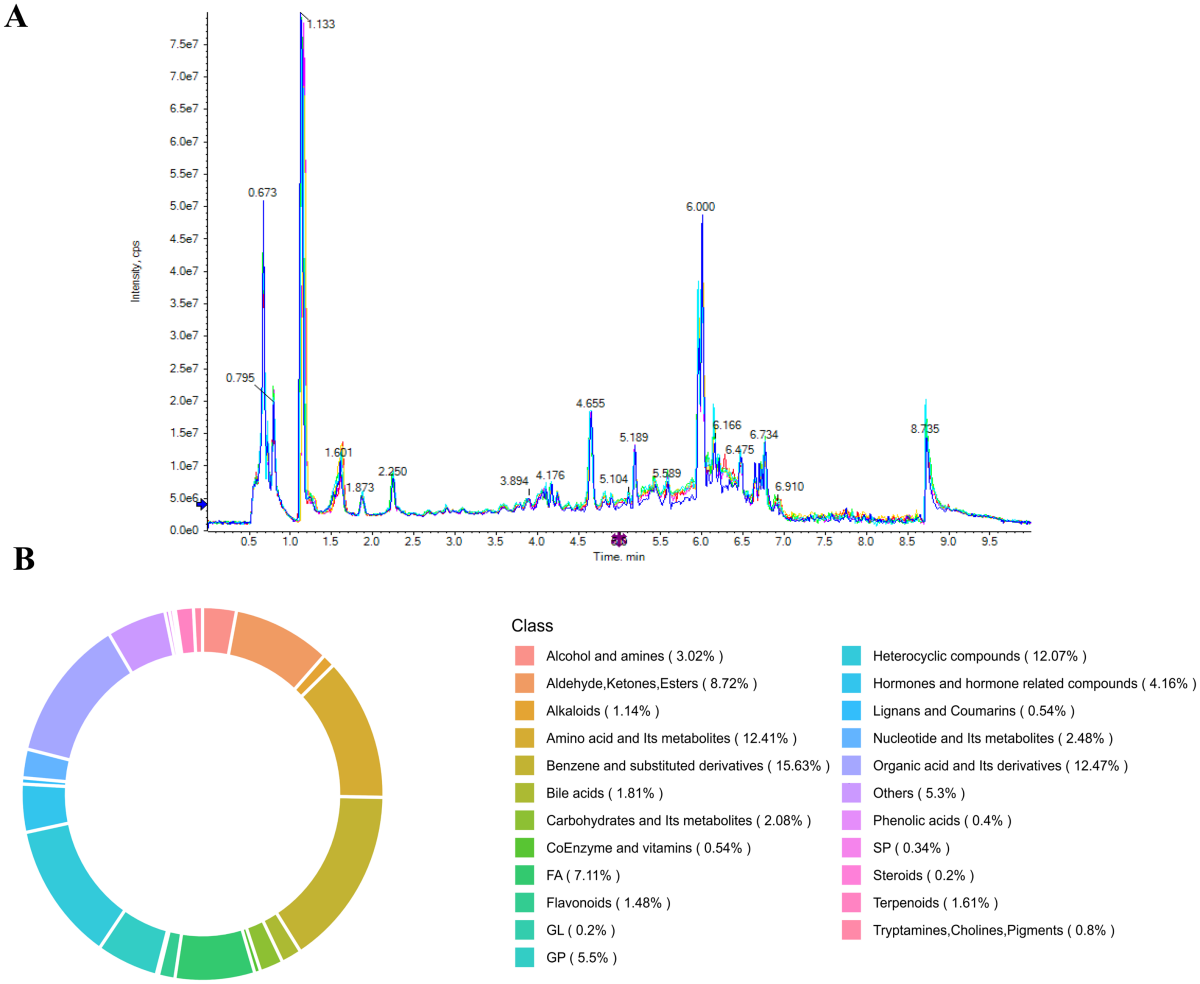
Serum metabolomic analysis of patients with ICH. **(A)** Total ion current chromatograms (TIC) of serum from patients with ICH. **(B)** Analysis of metabolite class proportions in serum from patients with ICH.

This study first analyzed the overall distribution of serum metabolites by LC–MS analysis of serum samples from patients with ICH(Figure 1). TIC analysis revealed multiple characteristic peaks of metabolites in ICH serum at retention times of 0.673, 0.795, 1.133, 6.000 and 8.735 min (Figure 1A). For the control group, characteristic peaks were observed at retention times of 0.654, 1.142, 2.256, 5.997 and 6.416 min (Supplementary Figure S1A), with the dominant peak at 6.416 min notably higher in intensity compared to the ICH group, suggesting differential abundance of lipid-related metabolites between the two groups. A total of 3,178 metabolites were identified in the serum of both groups. Identification of the compounds corresponding to the characteristic peaks of patients with ICH revealed that benzene and substituted derivatiyes accounted for the highest proportion (15.63%) (Figure 1B), followed by organic acids and their derivatives (12.47%), amino acids and their metabolites (12.41%), heterocyclic compounds (12.07%) and aldehydes/ketones/esters (8.72%). Benzene and substituted derivatiyes were found to be the most abundant which can suggesting that abnormalities in aromatic compounds may be associated with the pathology of ICH. In contrast, amino acids and their metabolites were the most abundant (18.52%) in control group, followed by benzene and substituted derivatiyes (14.40%), glycerophospholipids (GP, 9.93%) and organic acids and its derivatives (9.16%) (Supplementary Figure S1B). This study demonstrates that patients with ICH exhibit distinct serum metabolite profiles, with a marked increase in benzene and substituted derivatiyes.

### Data quality assessment of serum metabolomics

**Figure 2:**
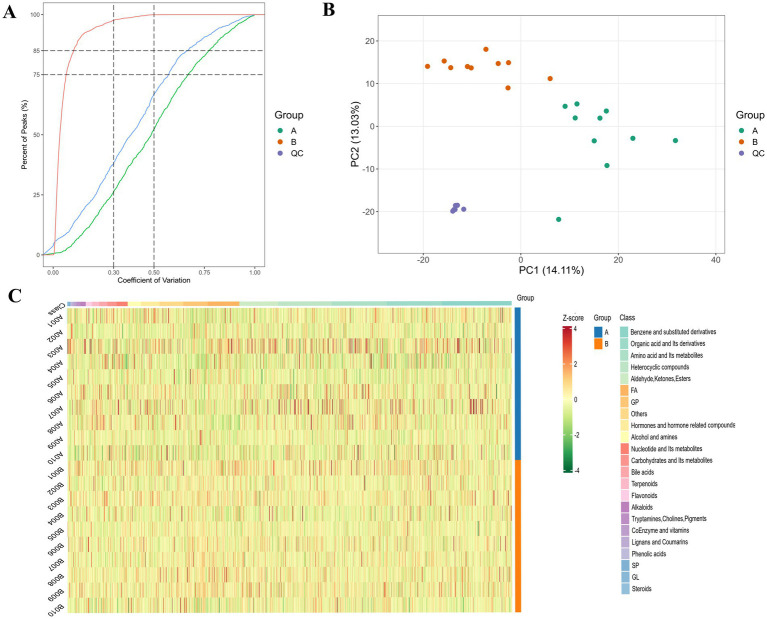
Stability and intergroup differences in serum metabolomics data between patients with and without ICH. **(A)** Cumulative distribution analysis of the coefficient of variation. **(B)** PCA analysis of serum metabolomics and intergroup differences between patients with and without ICH. **(C)** Cluster analysis of serum metabolite expression in patients with and without ICH. QC is quality control, A is the metabolome of patients with ICH, B is the metabolome of patients with and without ICH.

This study further evaluated the serum metabolomic profiles of patients with ICH using a multidimensional analysis system (Figure 2). Cumulative distribution analysis of the coefficient of variation (CV) (Figure 2A) showed that the coefficient of variation of metabolites in the QC group was significantly lower than that in the experimental group (CV < 0.3, accounting for >75%), indicating reliable data quality. The higher dispersion of Groups A and B (CV > 0.5, accounting for approximately 30%) may reflect disease heterogeneity. Principal component analysis (Figure 2B) revealed a clear separation along the PC1 axis (14.11%) in the ICH group (Group A), indicating significant metabolic reprogramming. The tight clustering of the QC group confirmed experimental stability. Difference heatmap analysis (Figure 2C) further demonstrated the specific enrichment of benzene and substituted derivatiyes, and amino acid metabolites in the ICH group (red blocks). These characteristic metabolites may serve as potential biomarkers. These studies confirm the unique metabolic profiles of patients with ICH which can provide new insights into disease mechanisms.

### Metabolomics analysis of OPLS-DA in patients with and without ICH

**Figure 3:**
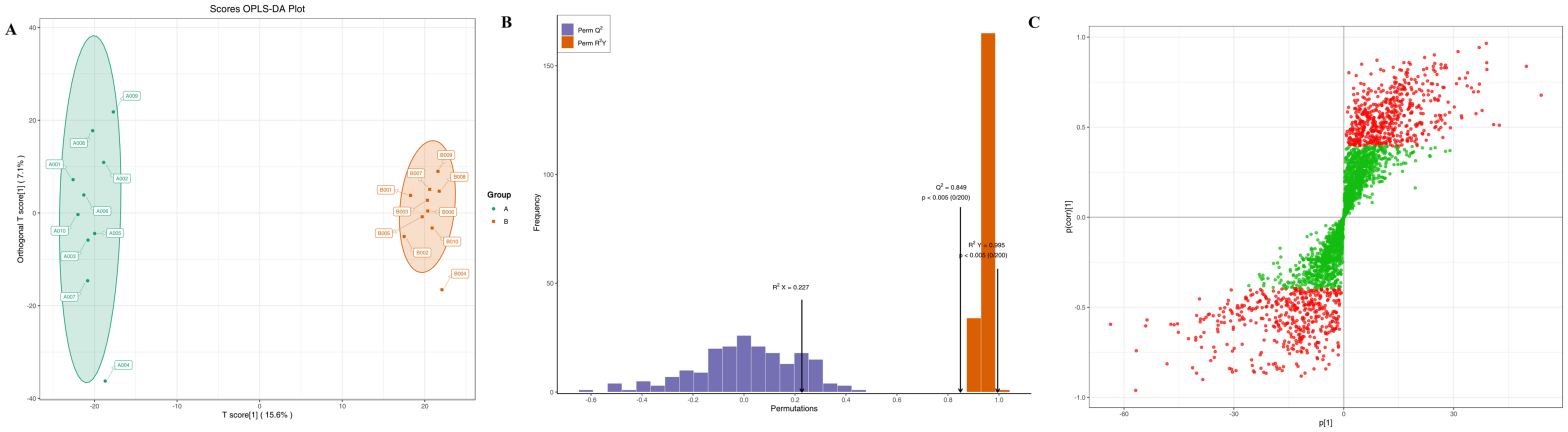
OPLS-DA model analysis of serum metabolomics in patients with amd without ICH. **(A)** OPLS-DA score plot of the serum metabolome of patients in the ICH and non-ICH groups. **(B)** OPLS-DA permutation test of the serum metabolome of patients in the ICH and non-ICH groups. The horizontal axis represents the model *R*^2^Y and *Q*^2^ values, and the vertical axis represents the frequency of model classification effects in 200 random permutation experiments. **(C)** OPLS-DA loading scatter plot of the serum metabolome of patients in the ICH and non-interICH groups. Red dots indicate metabolites with a VIP value greater than 1, and green dots indicate metabolites with a VIP value less than or equal to 1.

OPLS-DA was used to systematically evaluate the differences in serum metabolomics between patients with ICH (Group A) and patients without ICH (Group B) (Figure 3). The score plot (Figure 3A) showed that the two groups of samples were significantly separated along the t (1) axis (Group A was concentrated on the left and Group B was on the right), indicating that there were significant differences in the metabolic profiles of the two groups (*p* < 0.01). The permutation test (Figure 3B) further verified the robustness of the model. The *R*^2^Y (0.995) and Q^2^ (0.849) of the original model were significantly better than the random permutation results (*p* < 0.01), indicating that the differences between the groups were not caused by random factors. The VIP plot (Figure 3C) screened out metabolites that contributed significantly to the distinction between the groups (red points, VIP > 1). These characteristic metabolites were mainly enriched in the right area of the plot, which may be involved in key biological pathways (such as oxidative stress or energy metabolism). The OPLS-DA model can effectively distinguish the two groups of samples and provide a reliable statistical basis for the discovery of potential biomarkers.

### Differential metabolites in patients with and without ICH

**Figure 4:**
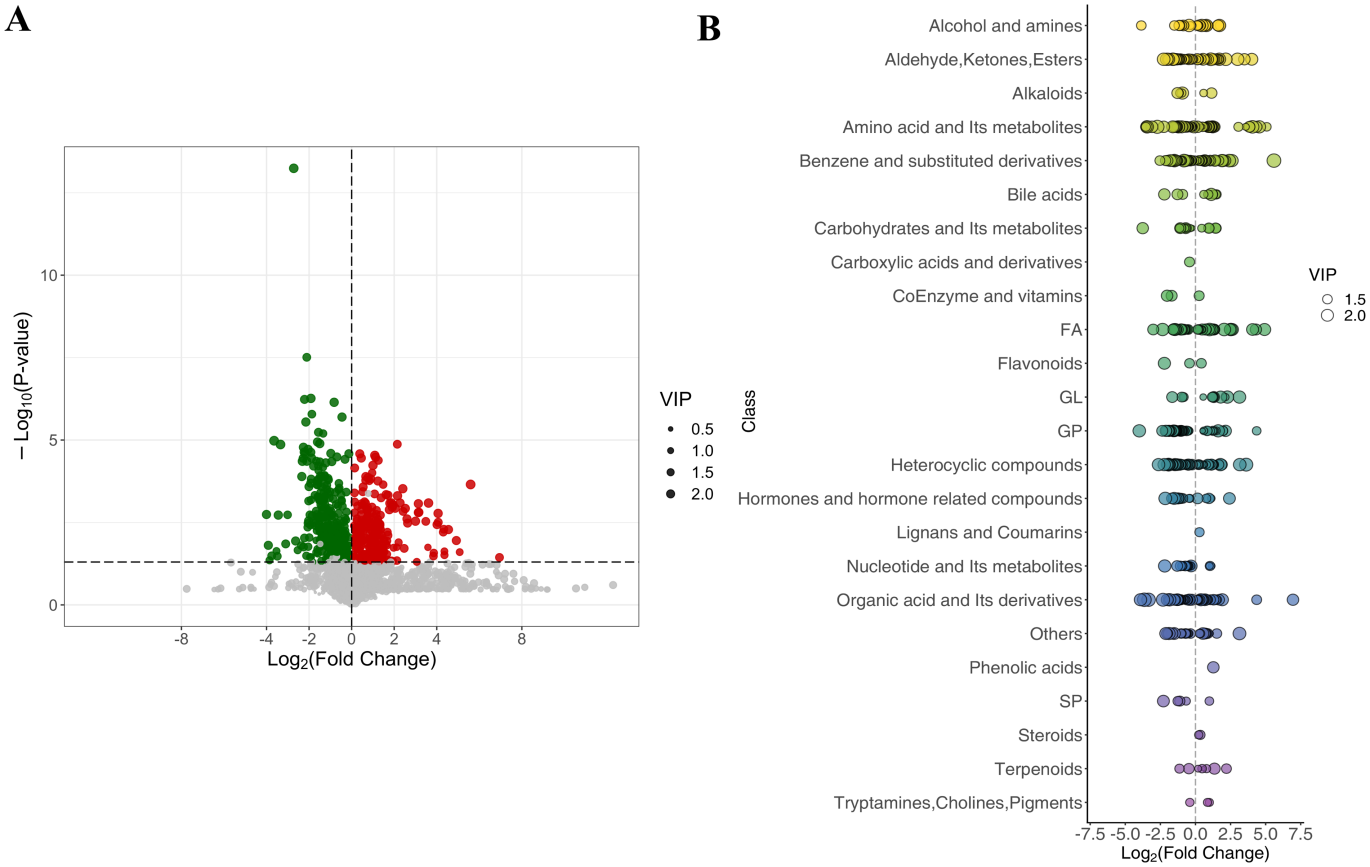
Differential metabolites in the serum metabolome of patients with and without ICH. **(A)** Volcano plot of differential metabolites in patients with and without ICH. **(B)** Classification of differential metabolites in patients with and without ICH.

This study compared the serum metabolomic profiles of patients with and without ICH to identify differentially expressed metabolites (Figure 4). The volcano plot (Figure 4A) revealed 651 significantly differentially expressed metabolites, of which 262 were significantly upregulated in the ICH group (red dots, log₂FC > 0), 389 were significantly downregulated (green dots, log₂FC < 0), and the remaining 2,141 metabolites showed no significant differences (gray dots). Among the upregulated metabolites, benzene and substituted derivatiyes (log₂FC = 3.2) and organic acids (log₂FC = 2.5) were the most significantly expressed, while amino acids and their metabolites showed a significant downregulation trend (log₂FC = −2.) (Figure 4B). Metabolite classification analysis further revealed that benzene and substituted derivatiyes comprised the largest proportion of differentially expressed metabolites and showed an overall upregulation trend, while lipids (such as FA, GP and SP) were generally downregulated. These results suggest that patients with ICH have obvious metabolic reprogramming characteristics, especially in the significant changes in aromatic compound metabolism and energy metabolism pathways.

### Enrichment analysis of differential metabolites in patients with and without ICH

**Figure 5:**
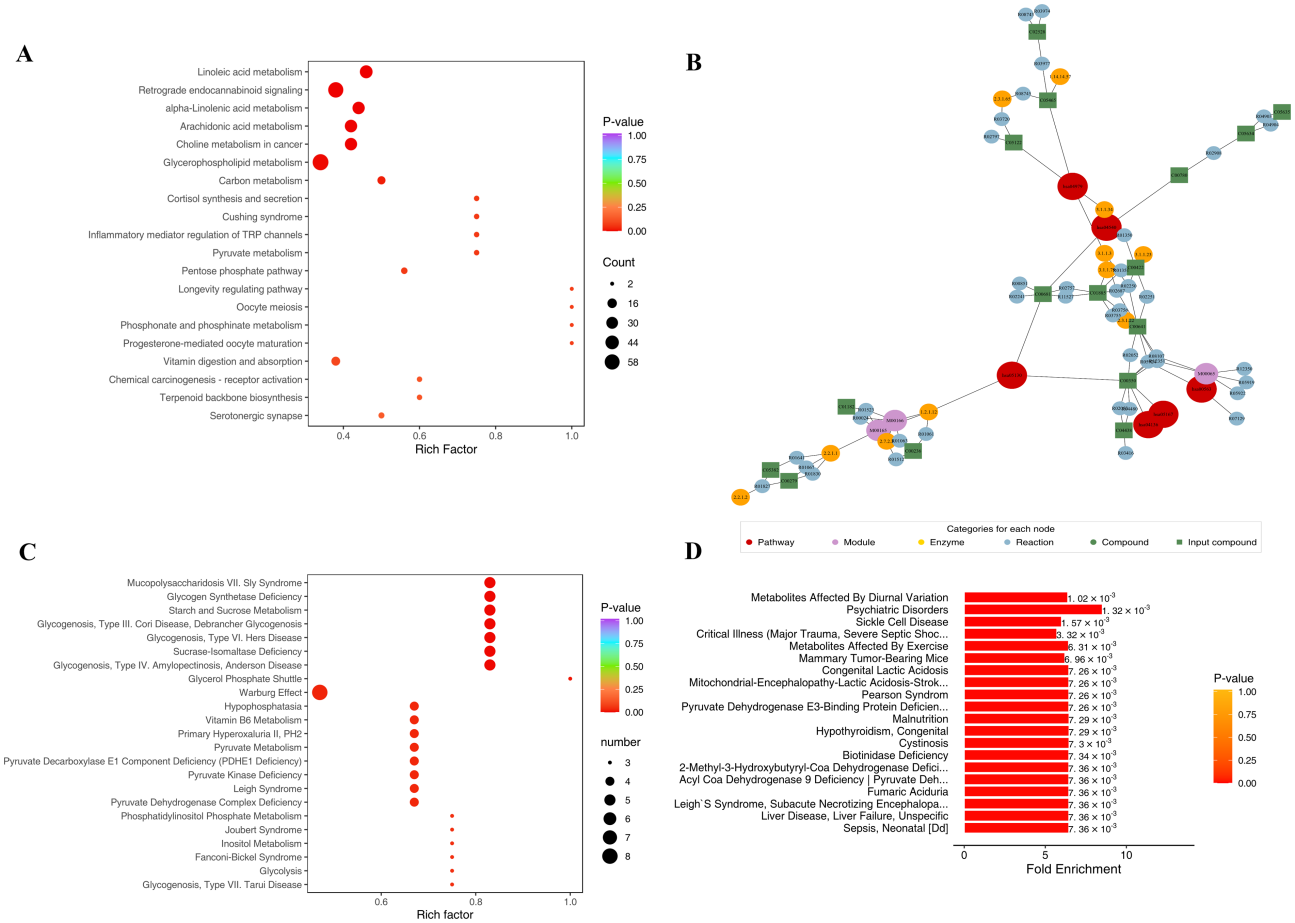
Enrichment analysis of differentially metabolites in the serum metabolome of patients with and without ICH. **(A)** KEGG pathway enrichment analysis of differential metabolites. **(B)** Multi-level regulatory network analysis of differential metabolites. The red dot is a metabolic pathway, the yellow dot is a substance-related regulatory enzyme information, the green dot is a background substance of a metabolic pathway, the purple dot is a class of substance molecular module information, the blue dot is a substance chemical interaction reaction, and the green square is the differential metabolite obtained in this comparison. **(C)** HMDB functional enrichment analysis of differential metabolites. **(D)** MSEA pathway enrichment analysis of differential metabolites.

This study systematically revealed key biological characteristics of differentially expressed metabolites between patients with and without ICH through multidimensional functional analysis (Figure 5). KEGG pathway enrichment analysis (Figure 5A) revealed that differentially expressed metabolites were primarily concentrated in linoleic acid metabolism, retrograde endocannabinoid signaling, alpha-linolenic acid metabolism, arachidonic acid metabolism, cancer-related choline metabolism, and glycerophospholipid metabolism, suggesting that abnormal lipid metabolism may be a key molecular hallmark of ICH. Co-occurrence network analysis (Figure 5B) constructed an association map between differentially expressed metabolites and illustrated that these metabolites are involved in multiple biological processes through complex molecular connections. Pathways related to inflammatory response and energy metabolism showed high connectivity, reflecting the systemic metabolic imbalance potentially characteristic of ICH. HMDB enrichment analysis (Figure 5C) further revealed that the differentially expressed metabolites were significantly associated with multiple metabolic diseases, including mucopolysaccharidosis VI (sly syndrome), glycogen synthetase deficiency, starch and sucrose metabolism, and glycogenosis, type III, suggesting that ICH may affect sugar and glycogen metabolism. Furthermore, MSEA enrichment analysis (Figure 5D) showed that the differentially expressed metabolites were enriched in metabolite sets associated with various physiological and pathological conditions, including metabolites affected by circadian variation, psychiatric disorders, sickle cell disease, metabolites affected by exercise and mammary tumor-bearing mice, and these findings are exploratory in nature and require further experimental validation. These findings provide new metabolic evidence for a deeper understanding of the pathophysiological mechanisms of ICH and may provide potential metabolic markers for clinical diagnosis and intervention.

### Diagnostic performance of key differential metabolites

**Figure 6:**
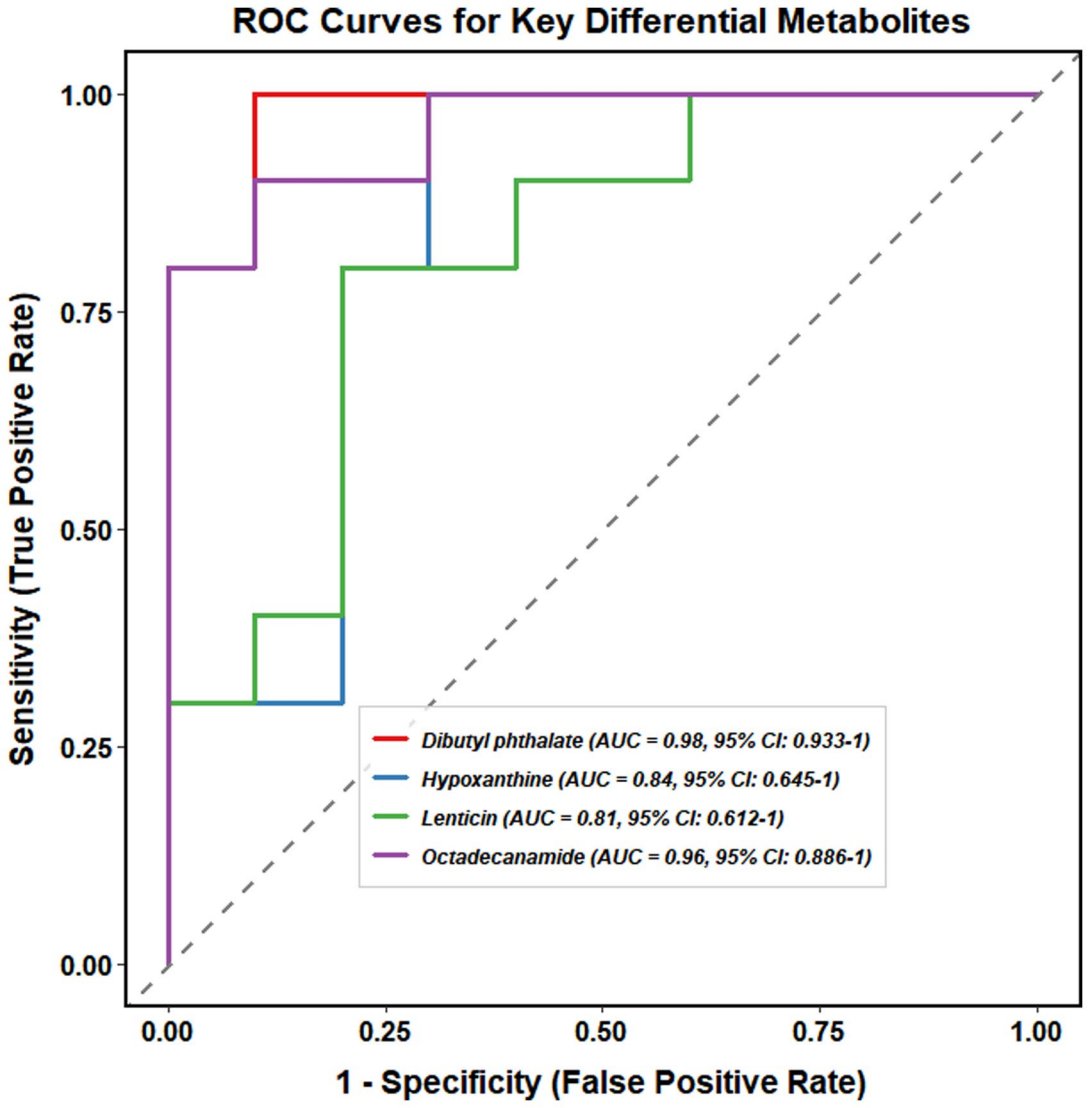
Receiver operating characteristic (ROC) curves of key differential metabolites for distinguishing ICH patients from healthy controls.

To rigorously cross-validate the multi-variable OPLS-DA model and assess the diagnostic capability of the specific biomarkers, univariate ROC analysis was conducted on the top four key differential metabolites (Figure 6). All four metabolites exhibited robust performance in discriminating ICH patients from healthy controls. Dibutyl phthalate and Octadecanamide demonstrated exceptional diagnostic accuracy, yielding AUC values of 0.980 (95% CI: 0.933–1.000) and 0.960 (95% CI: 0.886–1.000). Furthermore, Hypoxanthine (AUC = 0.840, 95% CI: 0.645–1.000) and Lenticin (AUC = 0.810, 95% CI: 0.612–1.000) also maintained high discriminatory capacity. These consistently high AUC values from the independent univariate analysis strongly validate that these single serum metabolites possess stable and reliable diagnostic potential, reinforcing the robust separation observed in our comprehensive metabolic profiling.

## Discussion

ICH is one of the most devastating types of stroke, characterized by high mortality and long-term disability (4). The one-year mortality rate for ICH exceeds 50%, and the majority of survivors suffer varying degrees of neurological impairment, such as hemiplegia, aphasia, dysphagia, cognitive impairment and dementia (33, 34). ICH not only reduces patients’ quality of life but also increases the costs of long-term care, rehabilitation and medical treatment. Fewer than 20% of patients are able to resume independent living within half year.

Clinically, the diagnosis and treatment of ICH primarily rely on imaging studies such as CT and MRI. However, these methods are limited by difficulties in patient transport, radiation exposure, and the inability to reflect molecular pathological processes (35). Therefore, the exploration of molecular biomarkers that can assist in stroke diagnosis, assess disease progression and predict prognosis is of great clinical significance. Metabolomics enables systematic, qualitative and quantitative analysis of small molecule metabolites within an organism which can comprehensively reflect its metabolic state (36). Metabolomics has been widely used in mechanistic research and biomarker discovery for a variety of diseases, including cardiovascular disease, diabetes, kidney disease and cancer (13, 37, 38).

There is increasing evidence that metabolic reprogramming plays a crucial role in the pathophysiology of ICH (39, 40). Cerebral vascular rupture and subsequent hematoma formation can cause mechanical damage and trigger severe biochemical disorders, such as oxidative stress, mitochondrial dysfunction, excitotoxicity and inflammatory responses (41, 42). Studies have found that the metabolomic characteristics associated with ICH include significant changes in the levels of multiple metabolites such as amino acids, carbohydrates, lipids and folic acid (43, 44). This is consistent with the abnormal metabolism of benzene and substituted derivatiyes, lipids, and amino acids in serum metabolites found in patients with ICH in this study. Systematic metabolic profiling provides new insights into disease mechanisms and also provides potential metabolic biomarkers for the early diagnosis and prognosis of ICH.

In this study, benzene and substituted derivatiyes were the most significantly upregulated, accounting for the highest proportion of differentially expressed metabolites. Aromatic compounds are intermediates in various endogenous and exogenous metabolic pathways, and their excessive accumulation is closely associated with oxidative stress, mitochondrial damage and neurotoxicity (45). Hematoma formation and erythrocyte lysis in ICH patients lead to iron overload and the production of reactive oxygen species (ROS). Abnormally elevated levels of aromatic compounds may enhance this oxidative environment, exacerbating lipid peroxidation and protein damage which in turn promotes neuronal necrosis and apoptosis (45). This phenomenon suggests that disturbances in aromatic compounds may not only be a consequence of ICH but may also play a role in pathological progression. Furthermore, this study observed a significant increase in organic acid metabolites. Organic acids are core components of energy metabolism and the tricarboxylic acid (TCA) cycle, and abnormal levels often reflect mitochondrial dysfunction and energy imbalance (46). During secondary ICH injury, a large number of neurons experience impaired mitochondrial function due to ischemia, hypoxia and oxidative stress which can lead to insufficient energy supply (47). Elevated serum organic acids indicate impaired glucose oxidation and increased anaerobic metabolism, which can exacerbate lactate accumulation and cellular acidosis (48). This metabolic imbalance not only affects neuronal survival, but also promotes excessive activation of glial cells by activating inflammatory signaling pathways (such as the NF-κB pathway), forming a vicious cycle and aggravating brain damage.

However, we found a general downregulation of amino acids and their metabolites, consistent with previous studies demonstrating excitatory amino acid depletion and neurotransmitter metabolism disturbances. Glutamate is the main excitatory neurotransmitter in the brain (49). In the early stage of ICH, it is released in large quantities due to the disruption of the blood–brain barrier and damage to the cell membranes. This massive release of glutamate leads to excitotoxicity and calcium overload, which can cause neuronal apoptosis (50). Furthermore, decreased depletion of glutamate and other amino acids further reflects an imbalance in neuronal energy metabolism and synaptic transmission (51). Disturbances in branched-chain amino acid (BCAA) metabolism may affect protein synthesis and energy replenishment, while alterations in tryptophan and its metabolic pathways are associated with neuroinflammation and depressive-like behaviors (52). Lipids are essential components of cell membranes and myelin sheaths and play key roles in energy storage, signal transduction and inflammation regulation (53, 54). Decreased lipid levels in ICH patients indicate damaged cell membrane structure and disrupted glycerophospholipid metabolism (55).

Differential metabolites in patients with ICH are primarily concentrated in key pathways that can reveal the molecular network underlying metabolic abnormalities in ICH, such as linoleic acid metabolism, arachidonic acid metabolism and glycerophospholipid metabolism. Both linoleic acid and arachidonic acid are polyunsaturated fatty acids, important components of cell membrane phospholipids and precursors of inflammatory signaling molecules (56). Following ICH, during hematoma formation and subsequent brain injury, phospholipase A2 (PLA2) activation leads to extensive hydrolysis of membrane phospholipids, releasing linoleic acid and arachidonic acid (57). This in turn generates a variety of inflammatory mediators, such as prostaglandins, leukotrienes and thromboxanes. These active lipid molecules can exacerbate localized brain edema and secondary neurological damage by regulating vasoconstriction, platelet aggregation, and inflammatory cell infiltration (58).

Several recent metabolomic studies on ICH have also identified key metabolites associated with cerebral hemorrhage. Chen et al. conducted multi-omics analysis combining serum untargeted metabolomics with gut microbiome profiling in ICH patients, identifying acylcarnitines (octanoylcarnitine, decanoylcarnitine, dodecanoylcarnitine), glyceric acid, and pyruvic acid as the most important diagnostic metabolites, with AUC values ranging from 0.872 to 0.932 (59). Wang et al. applied non-targeted LC–MS serum metabolomics to a mouse model of hypertensive intracerebral microhemorrhage, identifying 93 differentially expressed metabolites. Citrulline emerged as the most promising early biomarker candidate (AUC > 0.85), while the arginine and purine metabolic pathways showed the most significant alterations (60). Zhang et al. used metabolomics combined with an artificial neural network model to distinguish ICH from acute cerebral infarction, identifying 11 carnitine- and amino acid-based biomarkers with a sensitivity of 0.84 and specificity of 0.77 in an external test set (61). The aforementioned studies all indicate that disturbances in lipid, amino acid and energy metabolic pathways constitute a recurrent metabolic feature in ICH, this conclusion aligns with the findings of the present study.

Disturbances in glycerophospholipid metabolism also provide important clues to the pathological mechanisms of ICH. Glycerophospholipids form the skeleton of neuronal and mitochondrial membranes and play a role in signal transduction, vesicle trafficking and energy metabolism (62). This study observed a significant decrease in glycerophospholipid metabolites, indicating membrane damage and disrupted energy homeostasis. Previous studies have shown that blood–brain barrier disruption after ICH is closely associated with glycerophospholipid degradation, and degradation products such as lysophosphatidylcholine (LPC) can act as pro-inflammatory signals, activating microglia and exacerbating neuroinflammation (55, 58). Linoleic acid, arachidonic acid and glycerophospholipid metabolism are closely interconnected. Abnormal phospholipid metabolism provides substrates for fatty acid release, and fatty acid metabolites can also regulate membrane stability and inflammatory responses (63). This metabolic network disruption not only reveals the complexity of metabolic imbalances in ICH patients, but also suggests that inflammation and energy metabolism are highly coupled in the pathological evolution of ICH.

This study is subject to certain limitations. First, the sample size of this study is relatively small (*n* = 10). The strict exclusion criteria employed in this study were primarily designed to minimize the influence of metabolic confounding factors. In this single-center study, these criteria inevitably narrowed the scope of the eligible patient population, thereby contributing to the limited sample size. Second, due to the absence of an independent validation cohort, the candidate metabolites identified in this study should be regarded as exploratory findings rather than validated biomarkers. The discriminatory power of these metabolites requires prospective validation using targeted metabolomics approaches within larger, multi-center cohorts. Future studies should employ larger sample sizes and longitudinal study designs to validate the clinical relevance of the metabolic signatures identified herein, and to assess their potential utility in the diagnosis and prognostic assessment of ICH.

## Conclusion

This study comprehensively characterizes serum metabolic alterations associated with ICH. Upregulation of benzene and substituted derivatiyes and organic acids, downregulation of amino acid and lipid metabolites were found in ICH patients, indicating profound disturbances in aromatase metabolism, energy homeostasis and lipid signaling. Pathway analysis revealed that linoleic acid metabolism, *α*-linolenic acid metabolism, reverse endocannabinoid signaling, arachidonic acid metabolism, and glycerophospholipid metabolism are central to these metabolic disturbances, suggesting potential roles in inflammatory responses, neuronal injury, and recovery. Furthermore, several differentially expressed metabolites correlated with known metabolic disturbances and physiological states, providing candidate biomarkers for early detection, prognostic assessment, and therapeutic intervention. This study not only enhances our understanding of the biochemical landscape of ICH but also lays a theoretical foundation for future clinical therapeutic research targeting metabolic pathways.

## Data Availability

The datasets presented in this study can be found in online repositories. The names of the repository/repositories and accession number(s) can be found at: https://www.ebi.ac.uk/metabolights/, MTBLS13030.

## Footnotes

1 https://www.ebi.ac.uk/metabolights/, Number: MTBLS13030.

## Supplementary material

Supplementary Figure S1 Serum metabolomic analysis of patients without ICH. **(A)** Total ion current chromatograms (TIC) of serum from patients without ICH. **(B)** Analysis of metabolite class proportions in serum from patients without ICH.

Supplementary Table S1 Basic information of the study subjects.

Supplementary Table S2 Complete annotation and quantification data for all detected metabolite features.
