# Supplementary figures and images for: LC–MS-based serum metabolomics reveals distinct metabolic signatures in patients with intracerebral Hemorrhage
Source: Front Neurol. 2026 Jul 2;17:1795803. doi: 10.3389/fneur.2026.1795803 (PMC13372625; doi:10.3389/fneur.2026.1795803)

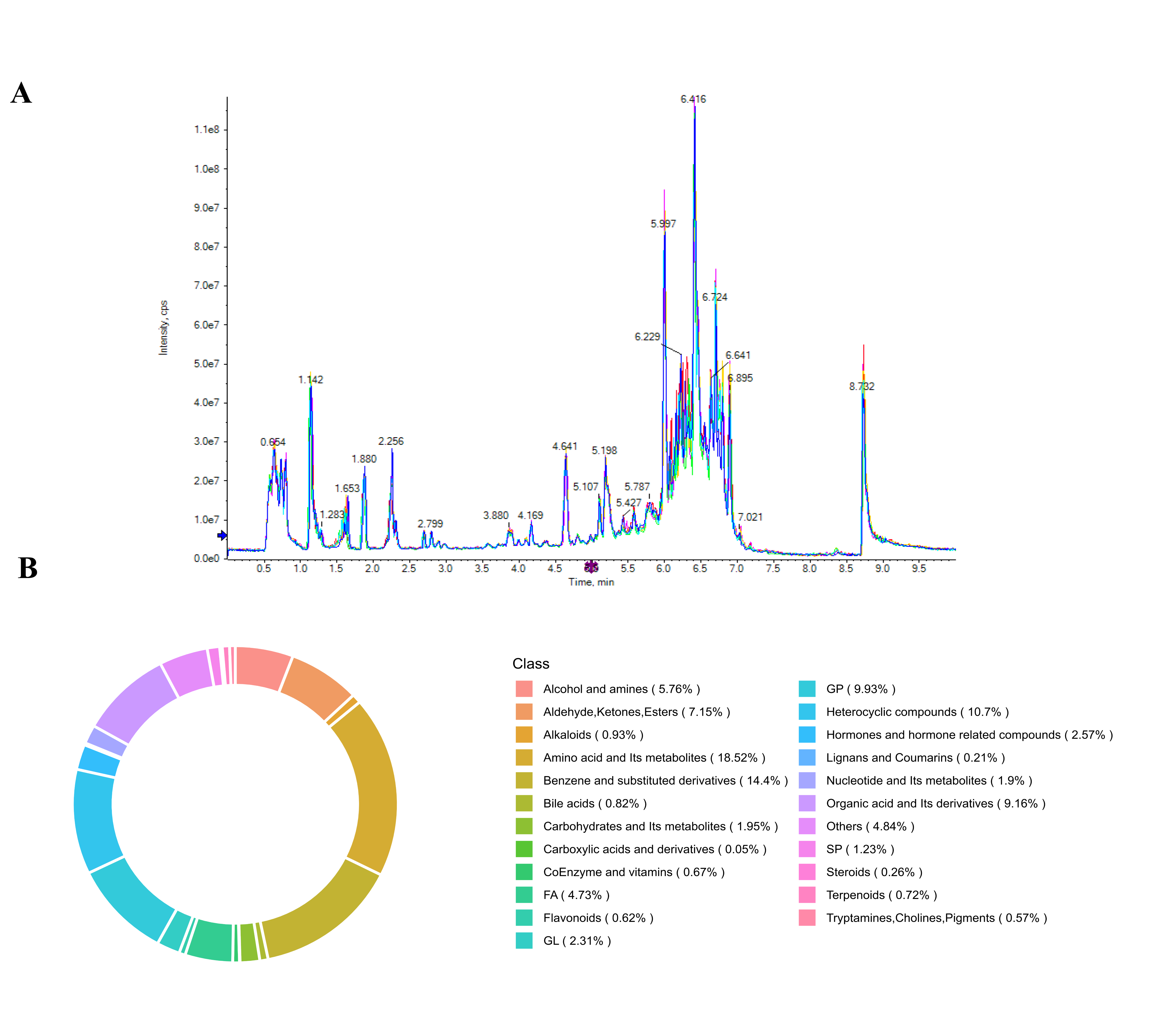

Supplement: Supplementary Figure S1 — Serum metabolomic analysis of patients without ICH. (A) Total ion current chromatograms (TIC) of serum from patients without ICH. (B) Analysis of metabolite class proportions in serum from patients without ICH. [file Image_1.TIFF]
